# Natural Pigment Production by *Bacillus velezensis* YM–3 Isolated from Traditional Pixian Douban Condiment: Biosynthesis Pathway, Structural Characterization, and Bioactivities

**DOI:** 10.3390/foods15122229

**Published:** 2026-06-20

**Authors:** Mamin Yue, Yanling Shang, Qing Zhang, Zihan He, Yu Qiu, Xiaomei Cheng, Qin Zhang, Wenliang Xiang, Jie Tang

**Affiliations:** 1 Food Microbiology Key Laboratory of Sichuan Province, School of Food and Bioengineering, Xihua University, Chengdu 610039, China; 2 Sichuan Advanced Agricultural & Industrial Institute–China Agricultural University, Chengdu 611430, China; 3 Chongqing Key Laboratory of Speciality Food Co–Built by Sichuan and Chongqing, Xihua University, Chengdu 610039, China

**Keywords:** *Bacillus velezensis*, pigment, biosynthesis pathway, structural characterization, characteristics

## Abstract

Natural microbial pigments offer important advantages and are widely studied for food applications. We investigated the biosynthetic pathways, characteristics, and bioactivities of the orange–red pigment produced by *Bacillus velezensis* YM–3, a strain isolated from the traditional Pixian Douban condiment. Whole-genome sequencing revealed complete pathways for melanin, phytoene, and heme biosynthesis. The purified extracellular pigment was characterized using ultraviolet–visible spectroscopy, Fourier-transform infrared spectroscopy, nuclear magnetic resonance spectroscopy, and ultra-performance liquid chromatography–high-resolution mass spectrometry; it was preliminarily characterized as melanin-like pigment. The pigment was highly soluble in alkaline solutions, moderately soluble in water, and insoluble in common organic solvents. It exhibited strong photostability and remained stable at low temperature, precipitated under acidic conditions, and showed high stability under alkaline environments. Furthermore, the pigment demonstrated in vitro free radical scavenging activity. Hence, this study provides a scientific foundation for exploring the potential utility of *B. velezensis* YM–3 and its pigment metabolites as functional agents.

## 1. Introduction

In the food industry, color is a crucial sensory attribute that directly influences consumer perception, purchase decisions, product quality evaluation, and, ultimately, commercial success, as consumers often associate specific colors with freshness, ripeness, flavor intensity, and the overall safety of food products. Consequently, pigments have become indispensable additives widely used across various industries, predominantly in food, followed by pharmaceuticals, cosmetics, and textiles [1]. Based on their origin, pigments can be classified as natural or synthetic [2]. Currently, synthetic pigments dominate industrial applications because of their low cost and high yields [3]. However, many synthetic pigments are toxic or carcinogenic, posing potential risks to human health, and their discharge as pollutants can also lead to ecological problems [4,5,6]. In contrast, natural pigments, primarily derived from microorganisms, plants, and animals, are generally considered safe for human consumption. Moreover, their environmentally friendly and biodegradable characteristics have increased their popularity among consumers [7,8,9].

Although pigment production has been reported in a wide range of bacteria, fungi, and actinomycetes, research in this domain remains at an early stage and requires further investigation [10]. For practical application, microbial strains intended for pigment production must meet key criteria, including high yield, nontoxicity, ease of purification, and, crucially, the sustainability and feasibility of the downstream extraction process [11]. Microbial pigments provide numerous advantages over those obtained from plants and animals, including lower production costs, shorter fermentation cycles, greater resilience to environmental fluctuations, ease of industrial scale-up, and broad application potential [10]. Nevertheless, many edible microbial pigments continue to undergo a slow translation to industrial use. Therefore, continued exploration of safe pigment-producing microorganisms from natural environments, particularly those associated with traditional fermented foods, is essential for identifying new pigment resources. Furthermore, developing optimized, green extraction strategies—based on the specific physicochemical properties of these pigments—is vital to bridge the gap between laboratory discovery and industrial feasibility.

Pixian Douban (PXDB) is a traditional Chinese semi-solid condiment with a long history. It is naturally fermented using *Aspergillus oryzae* as a starter culture and incorporates characteristic ingredients such as broad beans, chilies, wheat flour, and edible salt [12,13]. Traditional PXDB production involves an extended natural fermentation period following a “bask in daytime and dewed at night” process, resulting in a characteristic reddish-brown color that gradually deepens over time. A complex microbial community composed of diverse bacterial and fungal species plays a critical role in flavor development during natural PXDB fermentation [14]. Some of these microorganisms likely possess pigment–producing capabilities that contribute to the color evolution of PXDB. The contribution of microbial pigments to food color has been well documented in other fermented products. For instance, pigments produced by microorganisms colonizing cheese surfaces are important determinants of cheese color [15,16]. By analogy, screening pigment-producing strains from the distinctive microbial ecosystem of traditional PXDB and conducting in-depth investigations are of considerable interest. As a microorganism generally recognized for its probiotic potential, *Bacillis. velezensis* has been reported to improve the flavor of fermented foods [17,18]. However, pigment-producing *B. velezensis* strains have rarely been reported.

In this study, we isolated a pigment-producing strain, identified as *B. velezensis* YM–3, from traditional PXDB; elucidated its pigment metabolism; and conducted a comprehensive annotation of its genetic characteristics and metabolic pathways, determining the pigment type and clarifying its biosynthetic pathways. In addition, the fundamental physicochemical properties, stability, and in vitro antioxidant capacity of the pigments were evaluated. The rationale was to establish a foundational understanding of the *B. velezensis* pigment and to lay the groundwork for its potential application, together with that of the producing strain, in the food industry.

## 2. Materials and Methods

### 2.1. Source of Strain and Culture Medium

Target strains were screened from traditionally fermented PXDB. The culture media included YM medium (5 g/L peptone, 20 g/L agar, 3 g/L malt extract, 10 g/L glucose, and 3 g/L yeast extract) and a milk ingredient-based medium (5 g/L casamino acid, 5 g/L sodium chloride, 1 g/L potassium dihydrogen phosphate, 20 g/L glucose, 1 g/L yeast extract, and 20 g/L agar).

### 2.2. Screening and Identification of Pigment-Producing Microorganisms

PXDB samples (1 g) were homogenized and cultivated on YM or milk ingredient-based agar plates at 30 °C for 3 days. Visibly colored colonies were selected, isolated, and repeatedly purified to obtain single pure pigmented colonies.

The selected pigment-producing strains were cultivated on YM or milk ingredient-based medium for subsequent identification. Physiological and biochemical characterization was conducted according to the *Manual of Common Bacterial System Identification and Bergey’s Manual of Systematic Bacteriology*. Molecular identification was performed using 16S rRNA gene sequencing. A phylogenetic tree was constructed using MEGA software (version 7.0) to determine species affiliation.

### 2.3. Pigment Extraction and Yield Quantification

#### 2.3.1. Pigment extraction

Pigments were extracted using the method described by Zhang et al. [19]. Selected strains were inoculated at 2% (*v*/*v*) into YM or milk ingredient-based liquid medium and cultured at 30 °C for 3–5 days. Cells were harvested by centrifugation, and the precipitate was washed three times. The washed precipitate was homogenized in ultrapure water and disrupted using an ultrasonic homogenizer in an ice bath. The resulting cell disruption solution was extracted for 30 min and then centrifuged. The orange–red supernatant was collected and freeze-dried to obtain crude pigment. The pigment extraction efficiency was optimal at a solvent-to-biomass ratio of approximately 10–15 mL/g.

#### 2.3.2. Determination of Pigment Color Index and Yield

The color index, representing the ratio of complementary colors, was used to determine the dominant color component of the pigment solution. Absorbance was measured at 460, 510, and 610 nm using a UV–Vis spectrophotometer with ultrapure water as the blank. The red and yellow indices were calculated using the following formulas:

(1) $$\mathrm{red}\;\mathrm{index}\;=\;10\;\times \;\log_{10}(A_{510}/A_{610})$$

(2) $$\mathrm{yellow}\;\mathrm{index}=10\;\times \;\log_{10}{(A}_{410}/A_{610})$$

For yield determination, the selected strain was inoculated into 30 mL of medium. After cultivation and pigment extraction, the crude pigment extract was freeze-dried and weighed to calculate pigment yield.

### 2.4. Whole-Genome Sequencing and Functional Analysis

*B. velezensis* YM–3 was cultivated until the growth reached the mid-log phase, harvested by centrifugation at 13,400× *g*, and washed twice with sterile distilled water. Genomic DNA was extracted using a Tiangen Magnetic Bead DNA Extraction Kit (Tiangen Biotech, Beijing, China) [20]. DNA quantity, purity, and integrity were assessed using NanoDrop One spectrophotometry (Thermo Fisher Scientific, Waltham, MA, USA), a Qubit 4.0 Fluorometer (Thermo Fisher Scientific, Waltham, MA, USA), and 1.0% (*w*/*v*) agarose gel electrophoresis (requirements: OD260/280 = 1.8–2.0, total amount ≥ 10 μg, and concentration ≥ 50 ng/μL). Hig-molecular-weight DNA fragments (>10 kb) were selected using a BluePippin automated DNA recovery system (Sage Science, Beverly, MA, USA). Libraries were constructed using an SQK–LSK109 Ligation Sequencing Kit (Oxford Nanopore Technologies, Oxford, United Kingdom). Whole-genome sequencing was performed on a Nanopore sequencing platform (Beijing Biomarker Technologies Co., Ltd., Beijing, China). Filtered reads were assembled using Canu (v1.5), polished with Racon (v3.4.3), circularized, adjusted to the replication start site using Circlator (v1.5.5), and, finally, corrected using Pilon (v1.22) with second-generation reads.

Functional annotation was conducted using the Gene Ontology (GO), Kyoto Encyclopedia of Genes and Genomes (KEGG), carbohydrate-active enZYmes (CAZys), and evolutionary genealogy of genes: non-supervised orthologous groups (eggNOG) databases. The complete genome sequences were deposited in GenBank under accession numbers CP143843, CP143844, and CP143845.

### 2.5. Analysis of Fundamental Physicochemical Properties and Secretion Characteristics of the Pigment

Crude pigment powder (2 mg) was dissolved in 1 mL of different solvents (acetone, ethyl acetate, chloroform, methanol, ethanol, ultrapure water, HCl, NaOH, or ammonia) following a previously described method for pigment solubility [21]. Solubility was recorded after 30 min of incubation. Oxidizing agents (KMnO_4_, NaClO, H_2_O_2_, or K_2_Cr_2_O_7_) were added to the pigment solution, mixed thoroughly, and the bleaching effect was observed after 10 min. For the qualitative polyphenol test, crude pigment powder (10 mg) was dissolved in NaOH solution, and FeCl_3_ solution was added to observe color or precipitate formation.

Secretion characteristics were evaluated by inoculating *B. velezensis* YM–3 at 2% (*v*/*v*) into 100 mL of YM liquid medium and incubating at 30 °C with shaking at 180 rpm for 120 h. Samples were collected every 12 h and centrifuged (8000× *g*, 4 °C, 10 min) to separate supernatant and pellet. The pellet was sequentially washed with ultrapure water and 0.1 mol/L NaOH solution. The colors of the supernatant and washed pellets were recorded. Bacterial growth was monitored by measuring optical density at 600 nm (OD_600_). Pigments were extracted from samples at each time point, and yield was determined to assess correlation with bacterial growth.

### 2.6. Characterization of Pigment Structure

#### 2.6.1. Pigment Purification

Crude pigment powder was dissolved in 0.1 mol/L NaOH solution and sonicated. After centrifugation, the supernatant was collected, acidified to pH 1–2, and allowed to stand for 12 h. The precipitate was collected by centrifugation, rinsed to neutrality, and repeatedly washed with chloroform, ethyl acetate, and anhydrous ethanol. Finally, the precipitate was washed with ultrapure water until the supernatant tested negative for Cl^−^ ions, followed by centrifugation.

The purified precipitate was re-dissolved in NaOH solution and further purified using silica gel column chromatography (wet-packing method). The column was eluted with an ethanol gradient consisting of 200 mL each of absolute ethanol, 80%, 60%, 40%, 20%, and 0% (*v*/*v*) ethanol–water mixtures, in that order. Fractions exhibiting distinct colors were collected separately. The primary pigment components were freeze-dried, sealed, and stored.

#### 2.6.2. UV–Vis Spectroscopy

Pure pigment (0.1 mg) was dissolved in NaOH solution, and the absorption spectrum from 200 to 800 nm was recorded using a UV–Vis spectrophotometer (Shanghai Sunny Hengping Scientific Instrument Co., Ltd., Shanghai, China) to determine the maximum absorption wavelength.

#### 2.6.3. FT–IR Spectroscopy

A mixture comprising 2 mg of pigment and 200 mg of potassium bromide (KBr) was ground, pressed into a transparent pellet, and analyzed by FT–IR spectroscopy (PerkinElmer, MA, USA) over the range of 4000–400 cm^−1^ to identify major functional groups.

#### 2.6.4. NMR Spectroscopy

Purified pigment (20 mg) was dissolved in 1 mL of NaOD solution and analyzed by ^1^H NMR spectroscopy using a Bruker Avance DRX 600 spectrometer (Bruker BioSpin, Rheinstetten, Germany) at 25 °C.

#### 2.6.5. UPLC–HRMS Analysis

Pigment powder (10 mg) was dissolved in 300 mL of NaOH solution, vortexed for 60 s, and analyzed by UPLC. Conditions were as follows: Column: ACQUITY UPLC HSS T3 (2.1 mm × 100 mm, 1.8 µm; Waters, Milford, MA, USA); temperature: 40 °C; mobile phase A: 0.1% formic acid in water; B: acetonitrile; elution gradient: 5% B (0–1.5 min), to 10% B (1.5–2.5 min), to 40% B (2.5–14 min), to 95% B (14–22 min), hold at 95% B (22–25 min), to 5% B (25–26 min), hold at 5% B (26–30 min); flow rate: 0.4 mL/min; and injection volume: 5 µL.

MS and MS^2^ data were acquired using an Agilent 6545 Q–TOF mass spectrometer (Agilent Technologies, Santa Clara, CA, USA). Scan rates were 6 and 12 spectra/s for MS^2^. Scan ranges were *m*/*z* 50–1600 (MS) and *m*/*z* 20–1600 (MS^2^). ESI ion source settings were as follows: temperature, 320 °C; capillary voltage, 4000 V; nebulizer gas, 8 L/min; and sheath gas flow, 12 L/min.

### 2.7. Pigment Stability Studies

Pigment stability under various conditions was evaluated by measuring absorbance using a previously described method [22]. Light stability: Samples were exposed to outdoor sunlight, indoor incandescent light, UV light, or dark storage for 10 h. Thermal stability: Samples were incubated at 4, 25, 60, 80, and 100 °C for 12 h. pH stability: Pigment solutions were adjusted to pH 2.0–11.0 and maintained for 2 h. Metal ion stability: The influence of metal ions (Cu^2+^, Mg^2+^, Na^+^, Zn^2+^, Fe^2+^, K^+^, Ca^2+^, Al^3+^, and Mn^2+^) on the pigment solution was assessed after 2 h of incubation in the dark.

### 2.8. Pigment Antioxidant Effects

DPPH free radical scavenging activity was determined according to the method described by Safronova et al. [23]. Briefly, 1 mL of pigment solution was mixed with 1 mL of DPPH solution (0.1 mM), shaken, and incubated for 2 h. Absorbance was measured at 517 nm, using water instead of the pigment solution as the blank and anhydrous ethanol instead of the DPPH solution as the control.

Hydroxyl radical scavenging activity was measured as previously described [24]. A mixture containing 2 mL FeSO_4_ (6 mM), H_2_O_2_ (6 mM), and 2 mL pigment solution was allowed to stand for 10 min. Subsequently, 2 mL of salicylic acid solution (6 mM) was added, and the reaction was allowed to proceed for 30 min. Absorbance was recorded at 510 nm, with water replacing the pigment solution as the blank and H_2_O_2_ as the control.

ABTS free radical scavenging activity was evaluated according to the method described by Moghadam et al. [25]. The ABTS stock solution (7 mM) was mixed with an equal volume of K_2_S_2_O_8_ (4.9 mM) and incubated in the dark for 12–16 h. The working solution was diluted to an OD_734_ of 0.7. Subsequently, 10 μL of pigment solution was added to 200 μL of ABTS working solution and allowed to react in the dark for 5 min. Absorbance was measured at 734 nm, with water instead of the pigment solution as the blank and the ABTS working solution as the control. Scavenging activities against all three radicals were compared with those of Vitamin C, and antioxidant capacity was expressed as Vitamin C equivalents.

### 2.9. Statistical Analysis

All experiments were conducted in at least three independent biological replicates. Data are expressed as the mean ± standard deviation (SD). Normality of the data distribution was assessed using the Shapiro–Wilk test, and homogeneity of variance was evaluated using Levene’s test. Differences between groups were analyzed using one-way analysis of variance (ANOVA) followed by Tukey’s post hoc test to identify specific differences among means. Statistical significance was defined as *p* < 0.05. All statistical analyses were performed using IBM SPSS Statistics 27.0 (IBM, Armonk, NY, USA). Although the current results show high consistency across independent biological replicates, we acknowledge that the sample size (*n* = 3) provides preliminary precision. Further studies with expanded sample sizes are recommended to improve the robustness of these estimates against inherent biological variability.

## 3. Results and Discussion

### 3.1. Preliminary Screening of Pigment-Producing Microorganisms

**Figure 1:**
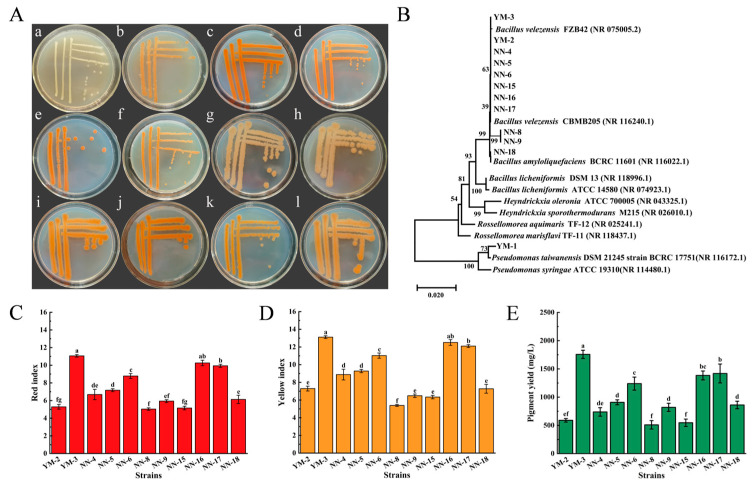
Screening and evaluation of pigment-producing strains isolated from Pixian Douban. (**A**) Colony morphology of the isolated pigment-producing strains: (**a**) YM–1; (**b**) YM–2; (**c**) YM–3; (**d**) NN–4; (**e**) NN–5; (**f**) NN–6; (**g**) NN–8; (**h**) NN–9; (**i**) NN–15; (**j**) NN–16; (**k**) NN–17; and (**l**) NN–18. (**B**) Phylogenetic tree constructed based on 16S rRNA gene sequences of the isolated strains. (**C**) Red index. (**D**) Yellow index. (**E**) Pigment yield of different strains. Different lowercase letters above the bars indicate significant differences (*p* < 0.05). Error bars represent the standard deviation (SD) of the measurements.

A total of 12 pigment-producing strains were isolated from PXDB, including three from the YM medium and nine from the milk ingredient-based medium. Colony characteristics are shown in Figure 1A. The colony morphology of strain YM–1 was milky yellow, round, opaque, and slightly convex at the center, with the color gradually faded from the center to the edges. The remaining 11 strains formed similar orange–yellow, round, opaque, dry, and wrinkled colonies (Figure 1A). The physiological and biochemical characteristics are listed in Table S1.

Molecular identification based on 16S rRNA gene sequencing and phylogenetic analysis (Figure 1B) indicated that strain YM–1 clustered with *Pseudomonas taiwanensis*, strain NN–18 clustered with *B. amyloliquefaciens*, and the remaining 10 strains clustered with *B. velezensis*. On the basis of morphological, physiological, biochemical, and phylogenetic evidence, YM–1 was identified as *P. taiwanensis*, NN–18 as *B. amyloliquefaciens*, and the others as *B. velezensis*.

### 3.2. Secondary Screening of Pigment-Producing Microorganisms

Strain YM–1 exhibited no significant pigmentation in liquid culture and was therefore excluded from further analysis. Pigments produced by the remaining 11 strains were quantified. Trends in the red index (Figure 1C), yellow index (Figure 1D), and pigment yield (Figure 1E) were consistent among strains. Strain YM–3 displayed the highest values, with a red index of 11.06 ± 0.04, a yellow index of 13.11 ± 0.04, and a pigment yield of 1757.61 ± 72.92 mg/L. Accordingly, strain YM–3 was selected for subsequent investigations.

### 3.3. Genomic Characteristics of B. velezensis YM–3

**Figure 2:**
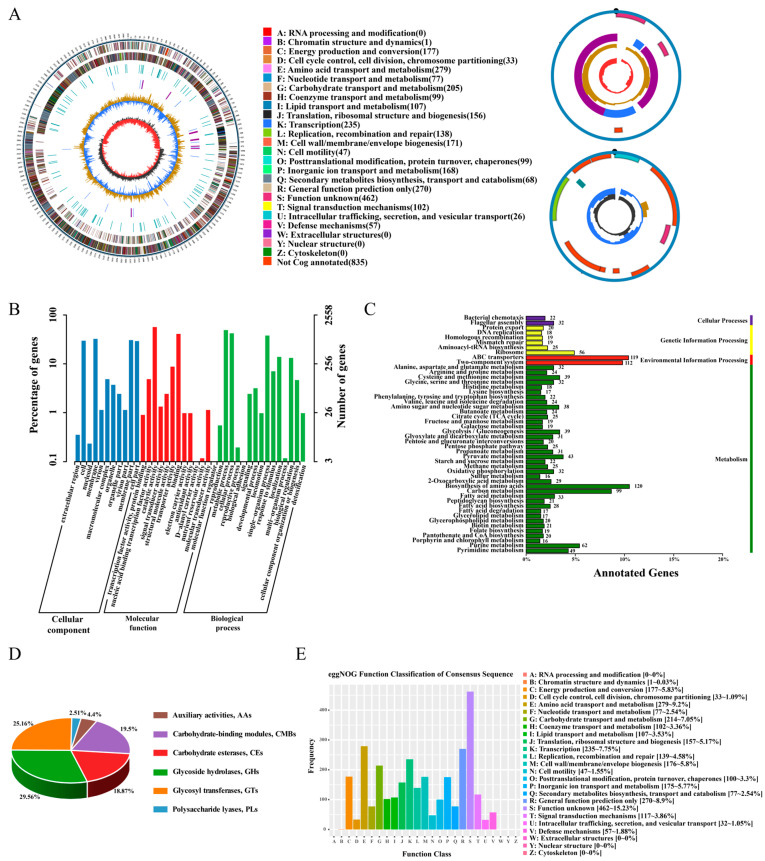
Genetic characterization of *B. velezensis* YM–3. (**A**) Circular genome and plasmid maps. (**B**) GO classification annotation. (**C**) KEGG classification annotation. (**D**) Distribution of carbohydrate-active enzymes. (**E**) Functional classification of eggnog identified functional genes.

The genome of *B. velezensis* YM–3 consisted of a circular chromosome (3,941,837 bp) and two plasmids (5993 bp; plasmid A, 8563 bp; plasmid B) (Figure 2A), totaling 3,956,393 bp with a GC content of 46.47% (Table S2). Genome annotation identified 3812 protein-coding genes (88% genome coverage) with an average length of 916 bp (range: 90–16,302 bp). Plasmids A and B encoded three and 10 genes, respectively. Noncoding elements included 30 rRNA genes (10 copies each of 5S, 16S, and 23S rRNA) and 96 tRNA genes.

The GO database provides a structured and continuously updated framework for the computational representation of gene and protein functions across diverse biological systems and species [26]. GO annotation assigned 2009 genes to molecular function (MF), 660 to biological processes (BPs), and 1328 to cellular components (CCs) (Figure 2B). KEGG is an integrated resource linking genomic, chemical, and systemic functional information to facilitate the molecular-level understanding of gene functions within biological pathways and networks [27]. KEGG analysis revealed that 2143 genes of *B. velezensis* YM–3 were enriched in 108 metabolic pathways, mainly involving the metabolism, genetic information processing, environmental information processing, and cellular processes. Notably, 120 genes were associated with amino acid biosynthesis, 99 with carbon metabolism, 119 with ABC transporters, and 112 with two-component systems, suggesting a strong metabolic capacity and extensive pathway utilization in *B. velezensis* YM–3 (Figure 2C).

The CAZy database is a specialized repository dedicated to carbohydrate-active enzymes [28]. CAZy annotation identified 140 genes, with glycoside hydrolases (GHs; 47 genes across 21 GH families; and 29.56%) and glycosyltransferases (GTs; 40 genes across 10 GT families; and 25.16%) representing the dominant categories. Prominent enzymatic functions were observed in the GH13 and GH126 families (five amylase-encoding genes) and the GH5 family (two cellulase-encoding genes). Additionally, 14 genes were assigned to the CBM50 family, which is related to peptidoglycan and chitin degradation (Figure 2D).

The eggNOG database comprises clusters of orthologous genes from diverse organisms and serves as an extension and refinement of the original COG database [29]. eggNOG annotation classified 2977 genes into 25 functional categories. The largest group corresponded to unknown function (Category S, 15.23%), followed by amino acid transport and metabolism (Category E, 9.2%), general function prediction only (Category R, 8.9%), transcription (Category K, 7.75%), carbohydrate transport and metabolism (Category G, 7.05%), energy production and conversion (Category C, 5.83%), and cell wall/membrane/envelope biogenesis (Category M, 5.8%). Metabolism-related genes accounted for 33.99% of the genome of *B. velezensis* YM–3 (Figure 2E), underscoring the metabolic versatility potentially supporting pigment production.

### 3.4. Genomic Assessment of B. velezensis YM–3 Safety

To evaluate the potential for industrial application, the genome of *B. velezensis* YM–3 was screened against the Virulence Factor Database (VFDB) (Table S3). No genes associated with known food-borne toxins (e.g., enterotoxins or emetic toxins) were identified. The presence of a few stress-survival and adherence-related genes is consistent with the strain’s adaptation to the fermented food environment rather than its pathogenic potential. Given its origin from traditional PXDB, strain YM–3 exhibits a favorable safety profile as a producer.

### 3.5. Prediction of Pigment Biosynthesis Pathways in B. velezensis YM–3

Whole-genome sequencing enables the prediction of a pigment’s biosynthetic pathways [30]. However, reports describing pigment biosynthesis genes and pathways in *B. velezensis* remain limited. Comprehensive genome-wide functional annotation of *B. velezensis* YM–3 revealed that this strain harbors candidate genes encoding enzymes associated with the biosynthetic routes of melanin, phytoene, and heme. However, it is important to note that the presence of these genes indicates the metabolic potential of the strain rather than their active expression under the experimental conditions described. Based on these genomic insights, putative biosynthetic pathways were inferred.

**Figure 3:**
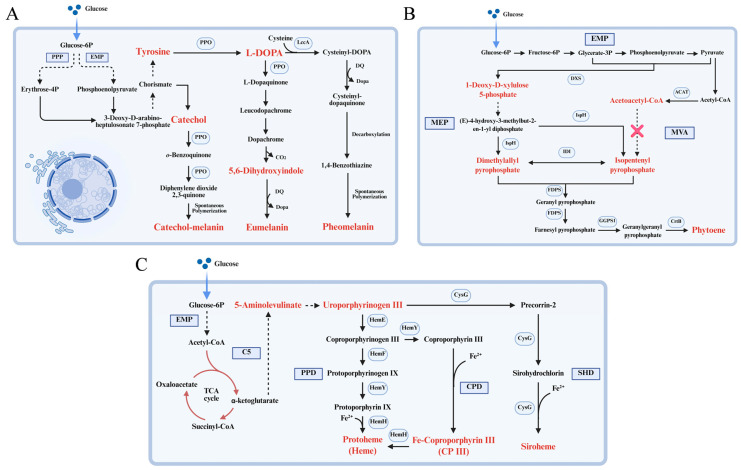
Predicted pigment biosynthetic pathways in *B. velezensis* YM–3 based on whole-genome analysis. (**A**) Melanin. (**B**) Phytoene. (**C**) Heme. Solid arrows indicate single-step reactions, whereas dashed arrows indicate multi-step reactions. ❌️ indicates the absence of the corresponding metabolic pathway. Detailed enzyme names and abbreviations are listed in Tables S3–S5. (PPP: pentose phosphate pathway; EMP: Embden–Meyerhof–Parnas pathway; MEP: methylerythritol phosphate pathway; MVA: mevalonic acid pathway; C5: C5 pathway; PPD: protoporphyrin-dependent pathway; CPD: coproporphyrin-dependent pathway; SHD: siroheme-dependent pathway; and L–DOPA: L–3,4–dihydroxyphenylalanine).

Melanin is a ubiquitous indole-derived pigment that includes eumelanin, pheomelanin, pyomelanin, and allomelanin. These pigments typically appear black or brown but can also exhibit red or yellow coloration [31]. Previous studies have identified three principal melanin biosynthetic pathways: the Raper–Mason, homogentisic acid (HGA), and dihydroxynaphthalene (DHN) pathways [32]. The Raper–Mason pathway, which utilizes tyrosine as a precursor, is commonly employed by bacteria to synthesize eumelanin and pheomelanin. Genomic analysis identified candidate genes required for tyrosine biosynthesis via the shikimate pathway (Table S4). Furthermore, two melanin biosynthesis routes were putatively predicted in *B. velezensis* YM–3 (Figure 3A). In one route, tyrosine is oxidized to L–3,4–dihydroxyphenylalanine (L–DOPA) by polyphenol oxidase (PPO), followed by the conversion of L–DOPA to cysteinyl–DOPA by laccase (lccA) in the presence of cysteine, after which decarboxylation, spontaneous oxidation, and polymerization generate pheomelanin. Alternatively, L–DOPA can be oxidized to L–dopaquinone by PPO and subsequently converted through a series of reactions into eumelanin. Catechol may also be directly oxidized and polymerized by PPO to form catechol-melanin.

Phytoene is an orange carotenoid synthesized from isopentenyl pyrophosphate (IPP) as a substrate and represents a key intermediate in lycopene biosynthesis. IPP is an intermediate metabolite in most organisms and can be synthesized via the methylerythritol phosphate (MEP) pathway in prokaryotes or the mevalonic acid (MVA) pathway in eukaryotes [33]. Functional annotation of the *B. velezensis* YM–3 genome (Table S5) revealed the presence of a complete MEP pathway and four enzymes potentially participating in phytoene formation: isopentenyl-diphosphate delta isomerase (IDI), farnesyl diphosphate synthase (FDPS), geranylgeranyl diphosphate synthase type III (GGPS1), and phytoene synthase (CrtB). Based on KEGG pathway analysis and genomic functional annotation, a phytoene biosynthetic pathway in *B. velezensis* YM–3 was proposed (Figure 3B). IDI mediates interconversion between IPP and dimethylallyl pyrophosphate (DMAPP). Subsequently, three IPP molecules and one DMAPP molecule are sequentially converted into geranyl pyrophosphate (GPP) and farnesyl pyrophosphate (FPP) via FDPS. FPP is then catalyzed by GGPS1 to form geranylgeranyl pyrophosphate (GGPP). Finally, condensation of two GGPP molecules by CrtB yields phytoene [34].

Heme is a pyrrole pigment formed by the complexation of ferrous ions with protoporphyrin IX (PPIX) and participates in light absorption, electron transfer, oxygen binding, and signal transduction, thereby playing central roles in essential processes such as respiration and photosynthesis [35]. The heme biosynthetic pathway is generally divided into three stages: synthesis of 5–aminolevulinate (5–ALA) via the C4 or C5 pathway, followed by the synthesis of the intermediate metabolite uroporphyrinogen III (UPG III) through a conserved core pathway, and conversion to heme via the protoporphyrin-dependent (PPD), coproporphyrin-dependent (CPD), or siroheme-dependent (SHD) pathways [36]. Functional annotation of the *B. velezensis* YM–3 genome identified 11 genes potentially encoding enzymes associated with the heme biosynthetic pathway (Table S6). Additionally, the genes encoding the CysG protein were present. CysG is a multifunctional protein capable of directly converting UPG III into heme [37]. As illustrated in Figure 3C, the putative heme biosynthetic pathway in *B. velezensis* YM–3 was inferred from KEGG mapping and functional annotation. α–ketoglutarate is converted into 5–ALA via the C5 pathway, subsequently transformed into UPG III through the core pathway, and finally directed toward protoheme, Fe–coproporphyrin, or siroheme through the PPD, CPD, and SHD routes, respectively. These genomic features suggest the potential of *B. velezensis* YM–3 to synthesize melanin, phytoene, and heme via multiple pathways, thereby providing a genetic foundation for future metabolic engineering and experimental verification.

### 3.6. Fundamental Physicochemical Properties of the Pigment

**Figure 4:**
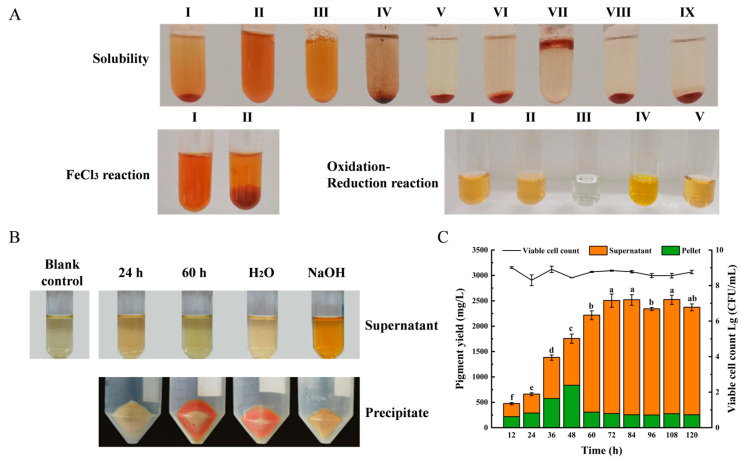
Physicochemical properties and secretion profile of the pigment produced by *B. velezensis* YM–3. (**A**) Solubility in various solvents (ultrapure water, NaOH, NH_4_OH, HCl, methanol, ethanol, chloroform, ethyl acetate, and acetone); reaction with FeCl_3_ solution (blank and test); and bleaching by oxidizing agents (H_2_O_2_, NaClO, K_2_Cr_2_O_7_, and KMnO_4_). (**B**) Determination of pigment secretion (intracellular or extracellular) during fermentation. (**C**) Time profile of pigment produced by *B. velezensis* YM–3. Different lowercase letters above the data points indicate significant differences (*p* < 0.05). Error bars represent the standard deviation (SD) of the measurements.

The physicochemical properties of *B. velezensis* YM–3 pigment are summarized in Figure 4A. The pigment was slightly soluble in water, soluble in alkaline solutions (NaOH and NH_4_OH), and insoluble in acidic solution (HCl) and common organic solvents (methanol, ethanol, chloroform, ethyl acetate, and acetone). The pigment was bleached by strong oxidants (H_2_O_2_, NaClO, and KMnO_4_). The addition of FeCl_3_ solution immediately produced reddish-brown flocculent precipitates, indicating a positive qualitative polyphenol reaction.

### 3.7. Pigment Secretion Profile

The pigment secretion profile of *B. velezensis* YM–3 is presented in Figure 4B. During the first 48 h of fermentation, the supernatant appeared red after centrifugation, whereas the pellet remained white. From 60 h onward, the supernatant resembled the original medium, while the pellet displayed a white base with a red surface layer. Washing the pellet with ultrapure water produced a light-red supernatant, accompanied by fading of the pellet color. Repeated washing resulted in a colorless supernatant. Subsequent treatment of the pellet with 0.1 mol/L NaOH generated a red supernatant and an almost white pellet. As shown in Figure 4C, pigment was predominantly present extracellularly in the supernatant during the initial 48 h. After 60 h, pigment precipitation was observed, with total yield reaching a maximum of 2521.64 ± 90.02 mg/L without significant change. These observations confirm that the pigment is an extracellular metabolite.

### 3.8. Pigment UV–Vis and FT–IR Spectral Analysis

**Figure 5:**
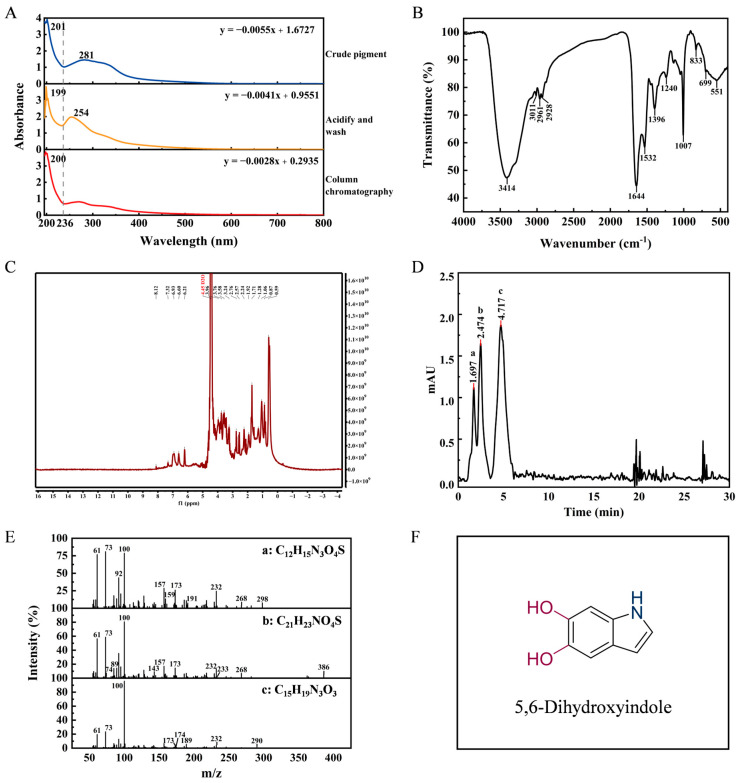
Structural characterization of the purified pigment from *B. velezensis* YM–3. (**A**) UV–Vis absorption spectrum. (**B**) FT–IR spectrum. (**C**) ^1^H–NMR spectrum. (**D**) UPLC profile. (**E**) HRMS spectrum. (**F**) Proposed tentative structural model of the core subunits of *B. velezensis* YM–3 pigment. The a, b, and c in Figure (**D**) correspond to a, b, and c in Figure (**E**), respectively.

The UV–Vis absorption spectra of *B. velezensis* YM–3 pigment obtained during purification are shown in Figure 5A. The crude extract exhibited peaks at 201 and 281 nm. The signal at 281 nm, characteristic of proteins and nucleic acids, suggested the necessity for further purification. Following purification, absorption in the 236–350 nm range decreased, confirming the removal of impurities. The purified pigment showed a maximum absorption at 200 nm, consistent with that of water-insoluble melanin isolated from *Streptomyces* sp. ZL–24 [38]. In the visible region, absorbance decreased with increasing wavelength, and the slope of the linear curve between the optical density and wavelength was −0.0028, characteristic of melanin [39].

FT–IR spectroscopy is widely applied for identifying functional groups in polysaccharides and pigments [40,41]. Characteristic vibrational frequencies of chemical bonds or functional groups generate absorption peaks at specific positions within the infrared absorption spectrum. FT–IR analysis (4000–450 cm^−1^) revealed multiple functional groups in the pigment (Figure 5B). A broad band near 3414 cm^−1^ corresponded to –OH or –NH stretching [42]. The peak at 3011 cm^−1^ indicated aromatic C–H stretching, whereas those at 2961 and 2928 cm^−1^ were attributed to alkyl C–H stretching [42]. Strong absorptions at 1644 and 1532 cm^−1^ were associated with benzene ring skeletal vibrations or, alternatively, with C=O stretching vibration and the N–H bending vibration of the amide bond, respectively [21]. The peak at 1396 cm^–1^ can be attributed to the bending vibration of methyl (–CH_3_). A sharp peak at 1007 cm^−1^ suggested C–O–C symmetric stretching. The absorption features in the 833–699 cm^−1^ region are indicative of aromatic ring substitution patterns leading to conjugated structures [43]. The absorption peak at 551 cm^−1^ may correspond to C–S stretching [39]. Collectively, these data support the presence of –OH/–NH, –CH_3_, –CH_2_, C–O–C, and possibly a benzene ring and amide groups in *B. velezensis* YM–3 pigment, while indicating the absence of C≡C, –CH(CH_3_)_2_, C≡N, or –C(CH_3_)_3_ groups, in agreement with reported melanin characteristics [21,43].

### 3.9. Pigment ^1^H–NMR Analysis

The ^1^H–NMR spectrum of the purified pigment (Figure 5C) displayed multiple signals in the chemical shift range δ 0.59–1.28 ppm, corresponding to alkyl C–H protons [44]. Peaks in the δ 2.24–3.96 ppm range were ascribed to –CH_2_– or –CH_3_ groups adjacent to nitrogen or oxygen atoms. The peak at δ 4.45 ppm corresponded to the residual D_2_O solvent. The signal at δ 6.21 ppm was assigned to the –NH proton of the indole moiety. Signals within the δ 6.60–7.32 ppm region are attributed to the aromatic hydrogen of the indole or pyrrole ring. The peak at δ 8.12 ppm may indicate the presence of a phenolic –OH group on the ring [43]. The absence of peaks beyond δ 10 ppm suggested a lack of free –COOH groups. Together, these observations indicate the presence of methyl, methylene, amine, and indole/pyrrole groups but not carboxyl groups, consistent with known features of melanin [43,44].

### 3.10. Pigment UPLC–HRMS Analysis

UPLC–HRMS fragment information can be used to infer molecular weights and the structural characteristics of unknown compounds [38]. The purified *B. velezensis* pigment produced three principal chromatographic peaks (Figure 5D). These peaks correspond to the major chemical substances enriched within the pigment and were analyzed accordingly to determine their structural characteristics.

Component a: The retention time was 1.697 min. A protonated molecule at *m*/*z* 298.0852 corresponded to the [M + H]^+^ ion. Major MS^2^ fragments included *m*/*z* 61.0192, 73.0554, 92.0285, 100.1017, 157.0058, 158.9626, 172.9782, 191.0733, 232.0280, 267.9582, and 298.0848 (Figure 5(Ea)). The molecular formula was deduced as C_12_H_15_N_3_O_4_S based on HRESIMS analysis (*m*/*z* 298.0852 [M + H]^+^, calculated for C_12_H_16_N_3_O_4_S^+^ *m*/*z* 298.0862, error = −3.35 ppm).

Component b: The retention time was 2.474 min. A protonated molecule at *m*/*z* 386.1430 corresponded to the [M + H]^+^ ion. Principal MS^2^ fragments were *m*/*z* 61.0229, 73.0587, 74.0622, 89.0518, 100.1034, 143.0247, 157.0041, 172.9751, 232.0204, 233.0223, 267.9467, and 386.1445 (Figure 5(Eb)). The molecular formula was deduced as C_21_H_23_NO_4_S based on HRESIMS analysis (*m*/*z* 386.1430 [M + H]^+^, calculated for C_21_H_24_NO_4_S^+^ *m*/*z* 386.1427, error = 0.78 ppm).

Component c: The retention time was 4.717 min. A protonated molecule at *m*/*z* 290.1487 corresponded to the [M + H]^+^ ion. Major MS^2^ fragments included *m*/*z* 61.0240, 73.0590, 100.1040, 172.9765, 173.9928, 189.0195, 232.0221, and 290.1488 (Figure 5(Ec)). The molecular formula was deduced as C_15_H_19_N_3_O_3_ based on HRESIMS analysis (*m*/*z* 290.1487 [M + H]^+^, calculated for C_15_H_20_N_3_O_3_^+^ *m*/*z* 290.1505, error = −6.20 ppm).

Integrated interpretation of the UV–Vis, FT–IR, ^1^H–NMR, and UPLC–HRMS results suggests that the pigment produced by *B. velezensis* YM–3 possesses structural motifs characteristic of sulfur-containing, carboxyl-free melanin-like substances. It should be noted that the precise structural elucidation of microbial melanin remains a formidable challenge due to its inherently polymeric, polydisperse, and heterogeneous nature. The analytical techniques employed in this study provided evidence for specific functional groups (e.g., indole/pyrrole rings, sulfur-containing groups, and alkyl chains) and an overall structural organization consistent with melanin. However, these methods are insufficient to fully determine the sequence of polymerization or the exact three-dimensional architecture of the macromolecule. Therefore, the proposed structure (Figure 5F) should be regarded as a plausible model representing the core building blocks—consistent with our spectroscopic data and biosynthetic potential identified via whole-genome analysis—rather than a definitive molecular identity.

### 3.11. Pigment Stability

**Figure 6:**
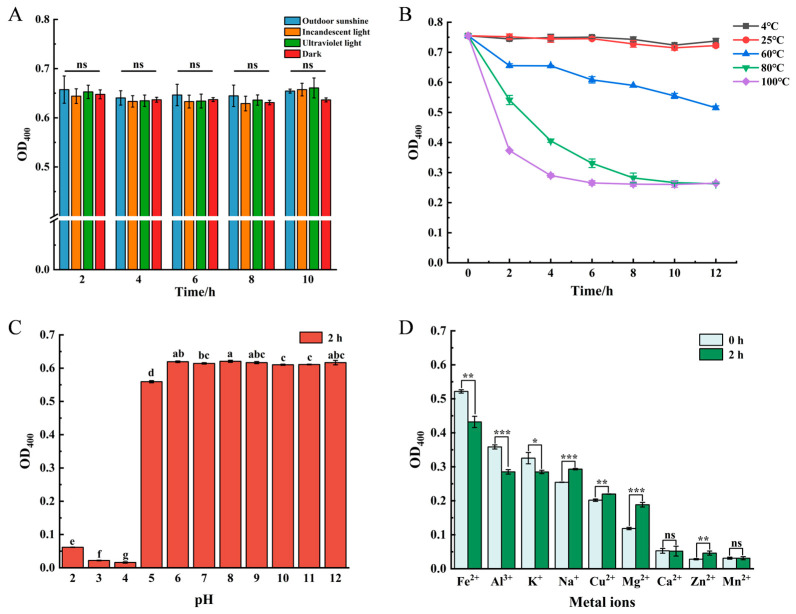
Stability of the *B. velezensis* YM–3 pigment under different environmental conditions. (**A**) Light exposure. (**B**) Temperature. (**C**) pH. (**D**) Metal ions. Different lowercase letters above the data points indicate significant differences (*p* < 0.05). Statistical significance is denoted as follows: ns, not significant (*p* > 0.05); *, *p* < 0.05; **, *p* < 0.01; and ***, *p* < 0.001. Error bars represent the standard deviation (SD) of the measurements.

Light stability is a crucial indicator for evaluating the stability of natural pigments. Many natural edible pigments undergo significant color changes when exposed to light. As shown in Figure 6A, no change in the absorption value of the pigment under the other three types of light irradiation compared with that under dark conditions. These results indicate that the pigment produced by strain YM–3 possesses good photostability and can resist light exposure while maintaining its original color and properties.

Temperature has a significant influence on pigment stability, and sensitivity to temperature is typically evaluated by the residual rate after treatment at different temperatures. As illustrated in Figure 6B, after incubation at 4 °C and 25 °C for 12 h, the pigment residue rates were 97.26% and 92.84%, respectively, indicating stability under these conditions. When treated at 60, 80, and 100 °C for 12 h, absorbance decreased significantly, with residue rates of 70.22%, 36.25%, and 35.30%, respectively. In summary, while the *B. velezensis* YM–3 pigment exhibits a certain degree of thermal stability, it is more appropriate for storage at low temperature.

The results for different pH pigment solutions (Figure 6C) showed that absorbance initially decreased, then increased, and finally stabilized across the pH range of 2.0–12.0. Significant precipitation occurred at pH 4.0, where the residue rate was only 2.64%. At pH > 5.0, the pigment residue rates exceeded 90% with no significant difference in absorption. These findings indicate that the pigment solution was more unstable under acidic conditions, whereas solubility and stability were higher under alkaline conditions. One possible explanation is that the pigment exists in ionic form in alkaline environments and that this ionic state becomes more stable as alkalinity increases.

When metal ions (Cu^2+^, Mg^2+^, Na^+^, Zn^2+^, Fe^2+^, K^+^, Ca^2+^, Al^3+^, and Mn^2+^) were added to the pigment solution, orange and red precipitates were formed following centrifugation. The supernatant was then used for absorbance measurement, and the results are presented in Figure 6D. Absorbance increased after the addition of Na^+^, Cu^2+^, or Mg^2+^ for 2 h, suggesting that these metal ions preserved the color and enhanced the pigmentation. In contrast, Fe^2+^ exerted the least effects on pigment stability; however, the residue rate remained only 72.85%. A possible explanation for precipitation induced by metal ions is that phenolic hydroxyl groups within the pigment, acting as multidentate ligands, chelate these ions and thereby form insoluble complexes.

### 3.12. Pigment Antioxidant Activity

**Figure 7:**
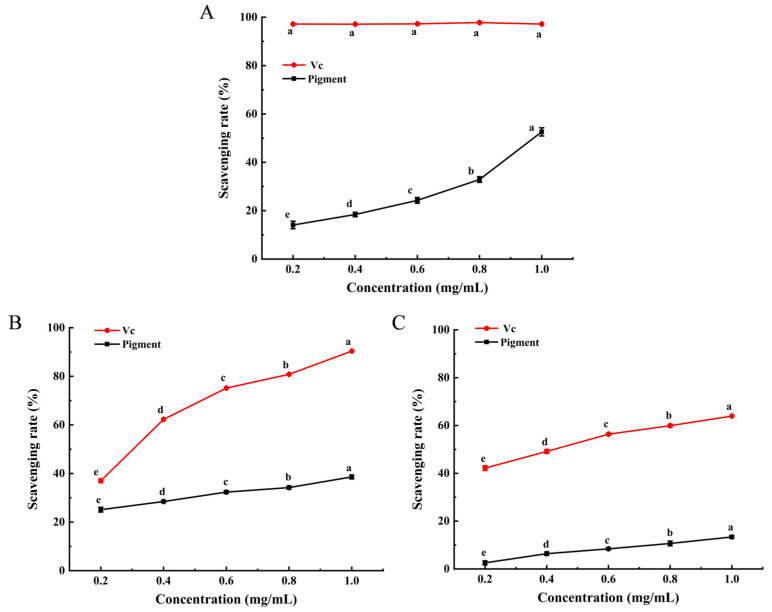
In vitro antioxidant properties of the *B. velezensis* YM–3 pigment. (**A**) DPPH. (**B**) Hydroxyl radical. (**C**) ABTS radical. Different lowercase letters above the data points indicate significant differences (*p* < 0.05). Error bars represent the standard deviation (SD) of the measurements.

DPPH radicals can accept electrons or hydrogen atoms to form stable molecules, and this reaction is widely employed to evaluate primary antioxidant capacity [45]. The scavenging activities of the pigment are shown in Figure 7A. DPPH radical scavenging increased as the pigment concentration increased. At a concentration of 1 mg/mL, the scavenging rate reached 52.59%.

Hydroxyl radicals are regarded as highly damaging reactive oxygen species capable of attacking various macromolecules in the human body [46]. Therefore, their removal is critical for antioxidant defense. As shown in Figure 7B, scavenging activity increased gradually, with increasing concentrations of pigment and Vitamin C within the range of 0.2–1.0 mg/mL; however, the increase was slower for the pigment. The maximum scavenging rate increased more slowly. The scavenging rate peaked at 38.59% when the pigment concentration reached 1 mg/mL. The ABTS radical scavenging rate is another important parameter for assessing antioxidant potential. Figure 7C shows that scavenging activity rose with the increasing pigment concentration. At 1 mg/mL, the ABTS radical scavenging rate was 13.38%.

Based on these results, the scavenging activities of *B. velezensis* YM–3 pigment against DPPH, hydroxyl, and ABTS radicals were concentration-dependent. Compared to Vitamin C, the scavenging capacity of 1 g of pigment was equivalent to 0.54 g, 0.43 g, and 0.21 g of Vitamin C for DPPH, hydroxyl, and ABTS radicals, respectively.

## 4. Conclusions

In this study, an orange–red pigment-producing strain, YM–3, was isolated from traditional PXDB and identified as *B. velezensis*. Genomic analysis predicted complete biosynthetic pathways for melanin, phytoene, and heme in *B. velezensis* YM–3. The pigment is an extracellular metabolite that is soluble in alkaline solutions, insoluble in common organic solvents, slightly soluble in water, and susceptible to bleaching by strong oxidants. Structural characterization demonstrated the presence of hydroxyl, amine, ether, methyl, methylene, and indole/pyrrole rings. These findings, combined with the absence of carboxyl groups and the presence of sulfur, suggest a unique molecular architecture. On the basis of predicted pathways, physicochemical properties, and structural features, the pigment was preliminarily classified as a melanin-like substance. The pigment exhibited strong photostability, suitability for storage at low temperature, precipitation under acidic conditions, enhanced stability in alkaline environments, and concentration-dependent antioxidant activity. These findings for *B. velezensis* YM–3 and its pigment establish a foundation for further investigation. Future research will prioritize comprehensive evaluations of toxicological safety, long-term stability within food systems, and broader functional performance to determine the practical feasibility of this pigment as a food additive.

## Data Availability

The original contributions presented in this study are included in the article/Supplementary Materials. Further inquiries can be directed to the corresponding author.

## Supplementary Materials

The following supporting information can be downloaded at: https://www.mdpi.com/article/10.3390/foods15122229/s1, Table S1: Physiological and biochemical test results of isolated pigment–producing strains. Table S2: The statistics of genome of strain YM–3. Table S3: The annotation of virulence factors of *B. velezensis* YM–3 in VFDBs. Table S4: The predicted genes of melanin biosynthesis pathway in *B. velezensis* YM–3. Table S5: The predicted genes of phytoene biosynthesis pathway in *B. velezensis* YM–3. Table S6: The predicted genes of heme biosynthesis pathway in *B. velezensis* YM–3.
