# Supplementary material for: Natural Pigment Production by Bacillus velezensis YM–3 Isolated from Traditional Pixian Douban Condiment: Biosynthesis Pathway, Structural Characterization, and Bioactivities
Source: Foods. 2026 Jun 20;15(12):2229. doi: 10.3390/foods15122229 (PMC13298460; doi:10.3390/foods15122229)
Supplement: Supplementary file 1 [file foods-15-02229-s001.zip › foods-4370834-supplementary.pdf]

Supplementary Table

Table S1. Physiological and biochemical test results of isolated pigment–producing strains

Strains	YM –1	YM –2	YM –3	NN –4	NN –5	NN –6	NN –8	NN –9	NN –15	NN –16	NN –17	NN –18
Gram stain	–	+	+	+	+	+	+	+	+	+	+	+
Starch hydrolysis	+	+	+	+	+	+	+	+	+	+	+	+
Cellulose decomposition	+	+	+	+	+	+	+	+	+	+	+	+
MR test	–	–	–	–	–	–	–	–	–	–	–	–
V–P test	+	–	+	+	–	+	+	+	+	+	+	–
Glucose	+	+	+	+	+	+	+	+	+	+	+	+
Sucrose	+	+	+	+	+	+	+	+	+	+	+	+
Inositol	+	+	+	+	+	+	+	+	+	+	+	+
Indol test	+	+	–	+	+	+	–	+	+	+	+	+
CAT test	+	+	+	+	+	+	+	+	+	+	+	+
Nitrate reduction	+	+	+	+	+	+	+	+	+	+	+	+
Citrate test	+	+	+	+	+	+	+	+	+	+	+	+

+ indicates positive; – indicates negative.

Table S2. The statistics of genome of strain YM–3

Feature	Value
Genome size (bp)	3,956,393
Number of contigs	3
Contig 1 length (bp)	5,993
Contig 2 length (bp)	3,941,837
Contig 3 length (bp)	8,563
Contig N50 (bp)	3,941,837
GC content (%)	46.47
Protein–coding genes	3,812
Coding sequence length (bp)	3,493,800
Mean gene length (bp)	916
Total repeat length (bp)	4,634
rRNA	30
tRNA	96

Table S3. The annotation of virulence factors of *B. velezensis* YM-3 in VFDB databases

Gene ID	VF ID	VF Gene	VF Category	Description
GE000876	VF0072	*clpC*	Stress survival	Endopeptidase Clp ATP-binding chain C
GE001576	VF0074	*clpP*	Stress survival	ATP-dependent Clp protease proteolytic subunit
GE001451	VF0141	*capC*	Immune modolation	CapC, involved in Poly-gamma-glutamate synthesis
GE001945	VF0411	*bslA*	Adherence	Hydrophobin BslA
GE002511	VF0560	*gnd*	Immune modolation	6-Phosphogluconate dehydrogenase

Table S4. The predicted genes of melanin biosynthesis pathway in *B. velezensis* YM–3

Number	Gene ID	Enzymes	Definition
1	GE002037	AroF, AroG, AroH	3–deoxy–7–phosphoheptulonate synthase
2	GE002644	AroB	3–dehydroquinate synthase
3	GE000191	AroD	3–dehydroquinate dehydratase I
4	GE000192, GE002332	AroE	shikimate dehydrogenase
5	GE000674	AroK, AroL	shikimate kinase
6	GE002654	AroA	3–phosphoshikimate1 carboxyvinyltransferase
7	GE000889, GE000890, GE001842, GE001948, GE002643	AroC	chorismate synthase
8	GE002645	AroH	chorismate mutase
9	GE002653	TyrA2	prephenate dehydrogenase
10	GE002652	HisC	histidinol–phosphate aminotransferase
11	GE000337	LccA	laccase
12	GE003274	PPO	polyphenol oxidase

Table S5. The predicted genes of phytoene biosynthesis pathway in *B. velezensis* YM–3

Number	Gene ID	Enzymes	Definition
1	GE002466	DXS	1–deoxy–D–xylulose–5–phosphate synthase
2	GE003156	DXR	1–deoxy–D–xylulose–5–phosphate reductoisomerase
3	GE000872	IspD	2–C–methyl–D–erythritol 4–phosphate cytidylyltransferase
4	GE000918	IspE	4–diphosphocytidyl–2–C–methyl–D–erythritol kinase
5	GE000871	IspF	2–C–methyl–D–erythritol 2,4–cyclodiphosphate synthase
6	GE002391	IspG	(E)–4–hydroxy–3–methylbut–2–enyl–diphosphate synthase
7	GE002382	IspH	4–hydroxy–3–methylbut–2–en–1–yl diphosphate reductase
8	GE001757, GE002482, GE003755	ACAT	acetyl–CoA C–acetyltransferase
9	GE002627	IDI	isopentenyl–diphosphate Delta–isomerase
10	GE002465	FDPS	farnesyl diphosphate synthase
11	GE000307, GE002465	GGPS1	geranylgeranyl diphosphate synthase, type III
12	GE003714	CrtB	phytoene synthase

Table S6. The predicted genes of heme biosynthesis pathway in *B. velezensis* YM–3

Number	Gene ID	Enzymes	Definition
1	GE0000022	ALT	alanine aminotransferase
2	GE000870	GluRS	glutamyl–tRNA synthase
3	GE002204	HemA	glutamyl–tRNA reductase
4	GE000121, GE002209	HemL	glutamate–1–semialdehyde 2,1–aminomutase
5	GE002208	HemB	porphobilinogen synthase
6	GE002206	HemC	hydroxymethylbilane synthase
7	GE002207, GE003583	HemD	uroporphyrinogen–III synthase
8	GE003775	HemE	uroporphyrinogen decarboxylase
9	GE000005, GE002348	HemF	coproporphyrinogen III oxidase
10	GE003773	HenY	protoporphyrinogen / coproporphyrinogen III oxidase
11	GE003774	HemH	protoporphyrin / coproporphyrin ferrochelatase
12	GE000666, GE003247, GE003248, GE003249	CysG	uroporphyrin–III C–methyltransferase / precorrin–2 dehydrogenase / sirohydrochlorin ferrochelatase
